# Supplementary material for: Copy number variation-associated lncRNAs may contribute to the etiologies of congenital heart disease
Source: Commun Biol. 2023 Feb 17;6:189. doi: 10.1038/s42003-023-04565-z (PMC9938258; doi:10.1038/s42003-023-04565-z)
Supplement: Supplementary file 2 — Supplementary Information [file 42003_2023_4565_MOESM2_ESM.pdf]

Supplementary Information for

Copy number variation-associated lncRNAs may contribute to the etiologies of congenital heart disease

Authors: Yibo Lu[1†], Qing Fang[1†], Ming Qi[1†], Xiaoliang Li[2], Xingyu Zhang[1], Yuwan Lin[1], Ying Xiang[1,3]*, Qihua Fu[1,3]*, Bo Wang[1,3]*

Affiliations:

[1]Pediatric Translational Medicine Institute, Shanghai Children's Medical Center, School of Medicine, Shanghai Jiao Tong University; Shanghai, China

[2]Department of Medical Genetics and Molecular Diagnostic Laboratory, Shanghai Children's Medical Center, School of Medicine, Shanghai Jiao Tong University; Shanghai, China

[3]Shanghai Key Laboratory of Clinical Molecular Diagnostics for Pediatrics; Shanghai, China

[†] These authors contributed equally to this work.

*Corresponding author. Email: booew@163.com; qfu@shsmu.edu.cn; xiangying@scmc.com.cn

Title page

The PDF file includes:

Supplementary Figure 1. Distribution of recurrent non-syndromic and syndromic CHD associated CNVs on human chromosomes.

Supplementary Figure 2. CNV-lncRNA coexpression modules mildly (|r| > 0.2) correlated to developmental stage.

Supplementary Figure 3. CHD associated CNV-lncRNA-miRNA-mRNA regulatory network.

Supplementary Figure 4. Principal component analysis of developmental heart and brain samples.

Supplementary Figure 5. Classification of CNV-lncRNAs in coexpression modules.

Supplementary Figure 6. Sequence conservation of CNV-lncRNAs in coexpression modules.

Supplementary Figure 7. Expression patterns of CNV-lncRNAs in heart-related non-syndromic black module during cardiomyocyte differentiation.

Supplementary Figure 8. Clustering of *HSALNG0104472* knockdown and overexpression samples.

Supplementary Figure 9. qPCR analyses of *HSALNG0104472* knockdown hiPSC-cardiomyocytes.

Supplementary Figure 10. Differentiation of *HSALNG0104472* knockdown human iPSCs to cardiomyocytes.

Supplementary Figure 11. Reduction of CNV-lncRNA *HSALNG0104472* may affect the efficiency of cardiomyocyte differentiation (Control group 1, including gating strategy).

Supplementary Figure 12. Reduction of CNV-lncRNA *HSALNG0104472* may affect the efficiency of cardiomyocyte differentiation (Control group 2).

Supplementary Figure 13. Reduction of CNV-lncRNA *HSALNG0104472* may affect the efficiency of cardiomyocyte differentiation (Control group 3).

Supplementary Figure 14. Reduction of CNV-lncRNA *HSALNG0104472* may affect the efficiency of cardiomyocyte differentiation (*HSALNG0104472* knockdown group 1).

Supplementary Figure 15. Reduction of CNV-lncRNA *HSALNG0104472* may affect the efficiency of cardiomyocyte differentiation (*HSALNG0104472* knockdown group 2).
Supplementary Figure 16. Reduction of CNV-lncRNA *HSALNG0104472* may affect the efficiency of cardiomyocyte differentiation (*HSALNG0104472* knockdown group 3).

Supplementary Table 1. Abbreviation and corresponding full names of CHD phenotype.
Supplementary Table 2. 21 CNVs associated with syndromic CHD.
Supplementary Table 3. Primers for quantitative reverse transcription qPCR analyses.
Supplementary Table 4. The silencer sequences for transient transfection in *HSALNG0104472* knockdown experiments.
Supplementary References.

Supplementary Figures

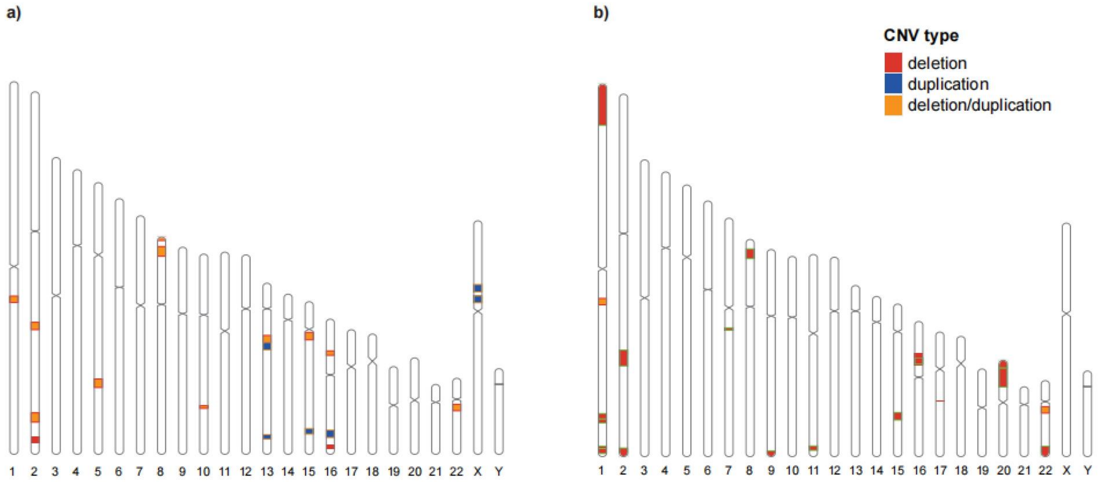

Supplementary Figure 1. Distribution of recurrent non-syndromic and syndromic CHD associated CNVs on human chromosomes. The colors of the bands represent CNV types. Totally 19 CNVs including 2 deletions, 6 duplications and 11 deletions/duplications were defined as recurrent non-syndromic CHD associated CNV **a)**. In comparison, most syndromic CHD associated CNVs (19/21) were deletions, the other 2 CNVs could be either deletion or duplication **b)**.

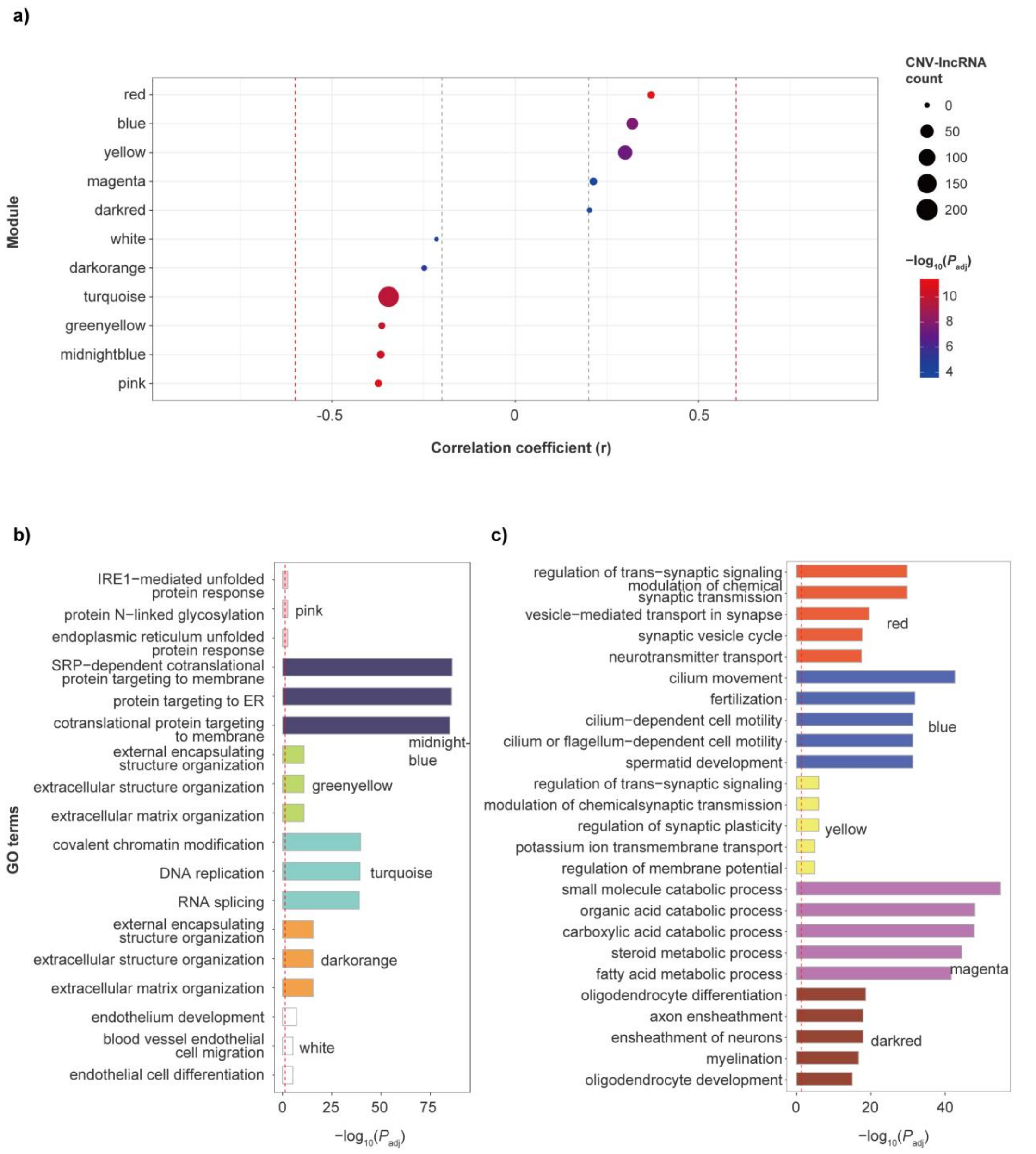

Supplementary Figure 2. CNV-lncRNA coexpression modules mildly (|r| > 0.2) correlated to developmental stage. Positively stage-correlated (r > 0.2, n = 5) and negatively stage-correlated coexpression modules (r < -0.2, n = 6) constructed with human organ developmental dataset (n = 313) are shown **a)**. The y axis represents different CNV-lncRNA coexpression modules. Values of correlation coefficient (r) to developmental stage are shown on the x axis. The grey dashed line indicates |r| = 0.2. The red dashed line indicates |r| = 0.6. Sizes of the nodes represent CNV-lncRNAs count in each module. Colors of the nodes represent values of $-\log_{10}(P_{adj})$. Functional annotation of negatively stage-correlated **b)** and positively stage-correlated **c)** coexpression modules are shown (Supplementary Data 2). Horizontal bars represent GO terms, and the colors of the bars represent different CNV-lncRNA coexpression modules. For each positively stage-correlated module, the top five GO terms (ranked by P_{adj}) are listed on the y axis. For each negatively stage-correlated module, the top three GO terms (ranked by P_{adj}) are listed on the y axis. Values of $-\log_{10}(P_{adj})$ are shown on the x axis. The red dashed line indicates P_{adj} of 0.05.

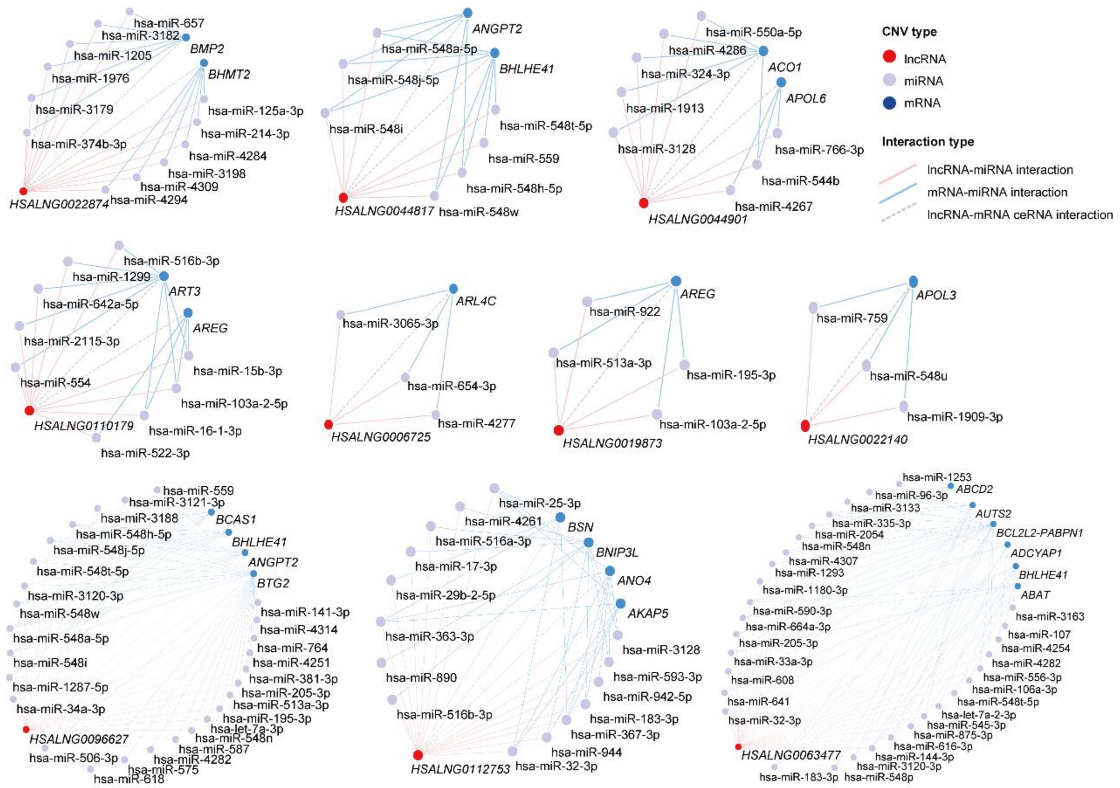

Supplementary Figure 3. CHD associated CNV-lncRNA-miRNA-mRNA regulatory network. Competing endogenous RNA (ceRNA) networks mediated by 10 hub CNV-lncRNAs are shown. Red dots represent CNV-lncRNAs. Blue dots represent mRNAs. Purple dots represent miRNAs. The red lines and blue lines represent lncRNA-miRNA and mRNA-miRNA interactions, respectively. The grey dashed lines represent ceRNA interactions.

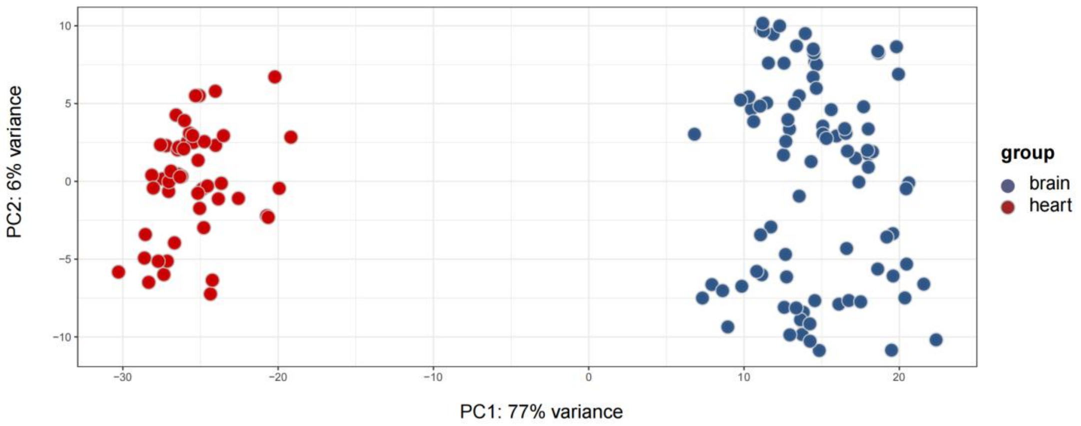

Supplementary Figure 4. Principal component analysis of developmental heart and brain samples.

Developmental heart samples (n = 50) and brain samples (n = 87) from human organ developmental transcriptomic data were clustered well. The colors of dots represent different tissue sources.

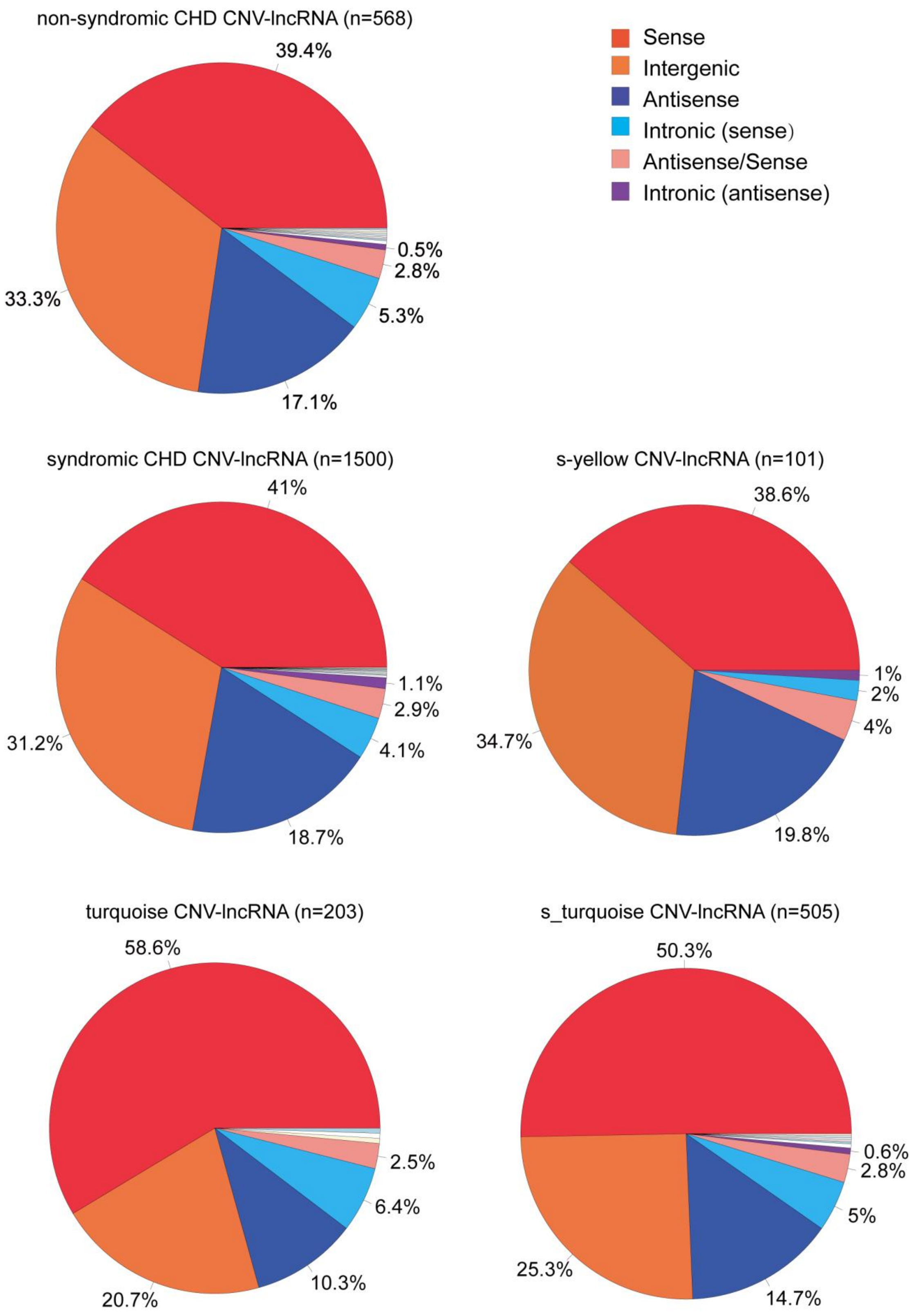

Supplementary Figure 5. Classification of CNV-lncRNAs in coexpression modules. Based on the genomic locations in respect to protein-coding genes, CNV-lncRNAs were classified into seven groups, Intergenic, Intronic sense, Intronic antisense, Overlapping sense, Overlapping (AS), sense, and Antisense (Supplementary Data 1). For each CNV-lncRNA, all types of transcripts were annotated and separated by slashes. See more details in Lncbook v2.0[1].

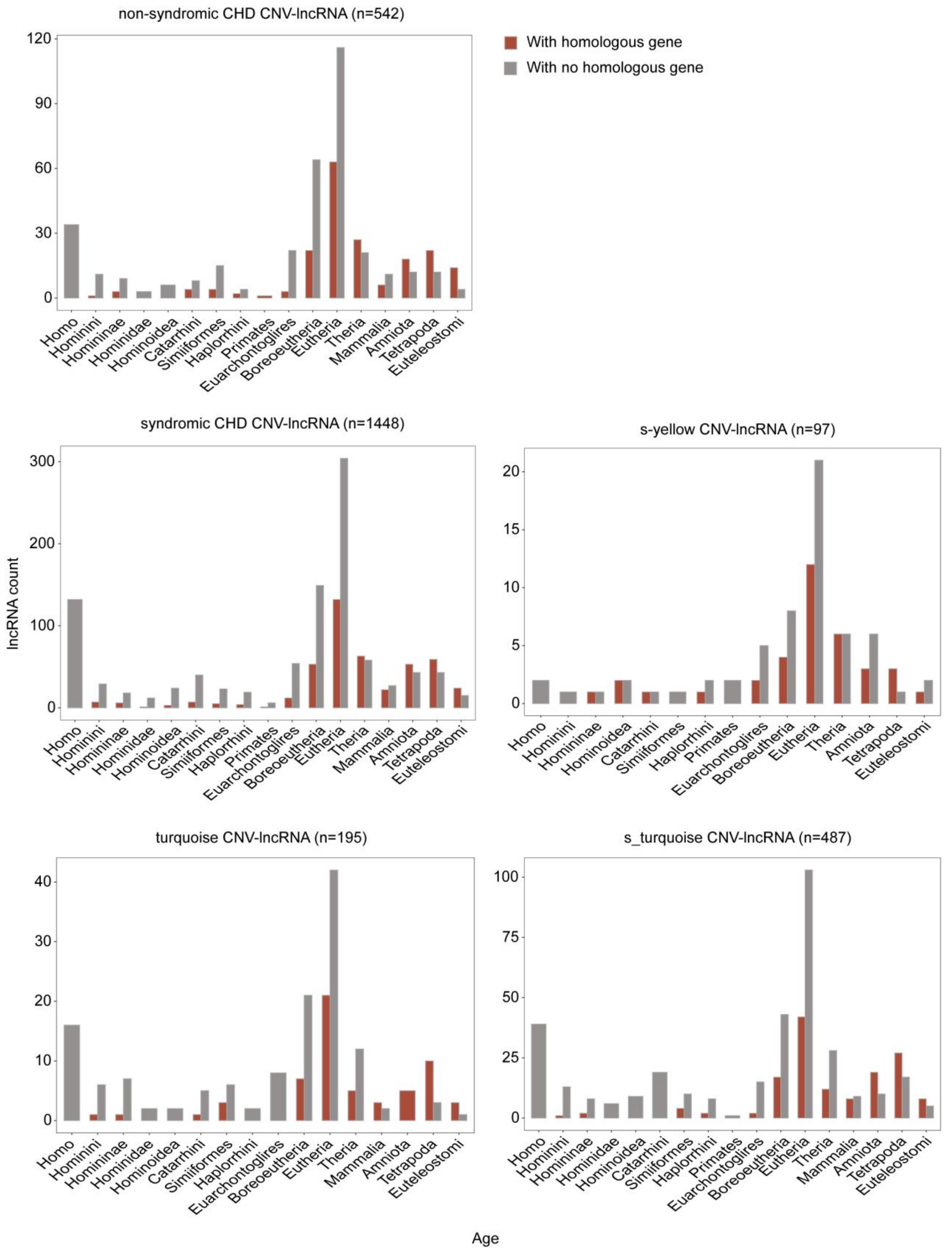

Supplementary Figure 6. Sequence conservation of CNV-lncRNAs in coexpression modules. Homologous protein-coding/non-coding genes and gene age for CNV-lncRNAs were identified based on the UCSC genome alignments between human and 40 vertebrates (Supplementary Data 3). Gene age of CNV-lncRNAs was defined as the earliest occurrence time of homologous sequence, which, from latest to earliest, are "Homo" (human specific), "Hominini", "Homininae", "Hominidae", "Hominoidea", "Catarrhini", "Simiiformes", "Haplorrhini", "Primates", "Euarchontoglires", "Boreoeutheria", "Eutheria", "Theria", "Mammalia", "Amniota", "Tetrapoda" and "Euteleostomi". The

time nodes correspond to the 17 important clades of the phylogenetic tree from zebrafish to human. See more details in Lncbook v2.0[1].

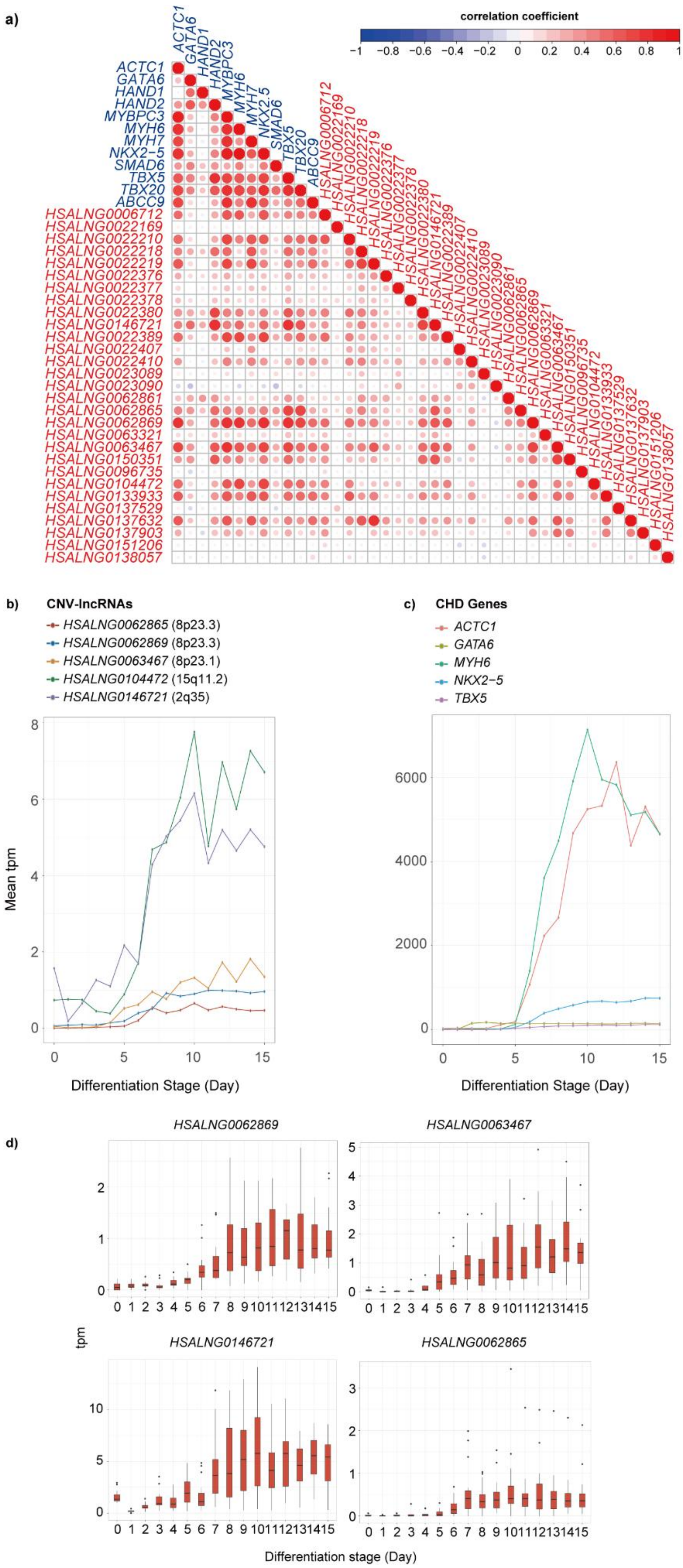
a)
correlation coefficient
-1 -0.8 -0.6 -0.4 -0.2 0 0.2 0.4 0.6 0.8 1
ACTC1
GATA6
HAND1
HAND2
MYBPC3
MYH6
MYH7
NKX2-5
SMAD6
TBX5
TBX20
ABCC9
HSALNG0006712
HSALNG0022169
HSALNG0022210
HSALNG0022218
HSALNG0022219
HSALNG0022376
HSALNG0022377
HSALNG0022378
HSALNG0022380
HSALNG0146721
HSALNG0022389
HSALNG0022407
HSALNG0022410
HSALNG0023089
HSALNG0023090
HSALNG0062861
HSALNG0062865
HSALNG0062869
HSALNG0063321
HSALNG0063467
HSALNG0150351
HSALNG0096735
HSALNG0104472
HSALNG0133933
HSALNG0137529
HSALNG0137632
HSALNG0137903
HSALNG0151206
HSALNG0138057
b)
CNV-lncRNAs
HSALNG0062865 (8p23.3)
HSALNG0062869 (8p23.3)
HSALNG0063467 (8p23.1)
HSALNG0104472 (15q11.2)
HSALNG0146721 (2q35)
Mean tpm
Differentiation Stage (Day)
c)
CHD Genes
ACTC1
GATA6
MYH6
NKX2-5
TBX5
Differentiation Stage (Day)
d)
HSALNG0062869
HSALNG0063467
HSALNG0146721
HSALNG0062865
tpm
Differentiation stage (Day)

Supplementary Figure 7: Expression patterns of CNV-lncRNAs in heart-related non-syndromic black module during cardiomyocyte differentiation. **a)** Pearson correlation coefficient (calculated with in vitro cardiomyocyte differentiation dataset, n = 297) between CNV-lncRNAs and 12 CHD genes in the black module are shown. Sizes of dots represent the absolute value of corresponding correlation coefficient. The colors of dots represent the value and direction of correlation (red: positive, blue: negative). Besides *HSALNG0104472* (shown in Fig. 7b), expression patterns of other four CNV-lncRNAs that significantly correlated to CHD genes ($r > 0.7$ and adjusted P value < 0.05, Supplementary Data 11) in heart-related black module and five key CHD genes are shown in **b-d)** (A supplement to Fig. 7b). The x and y axes represent cardiomyocyte differentiation stage (day) and mean expression value (tpm) of each stage, respectively. The center line represents median. The box limits represent upper and lower quartiles. The whiskers represent 1.5x interquartile range. The points represent outliers. The P values were adjusted using the Benjamini-Hochberg method.

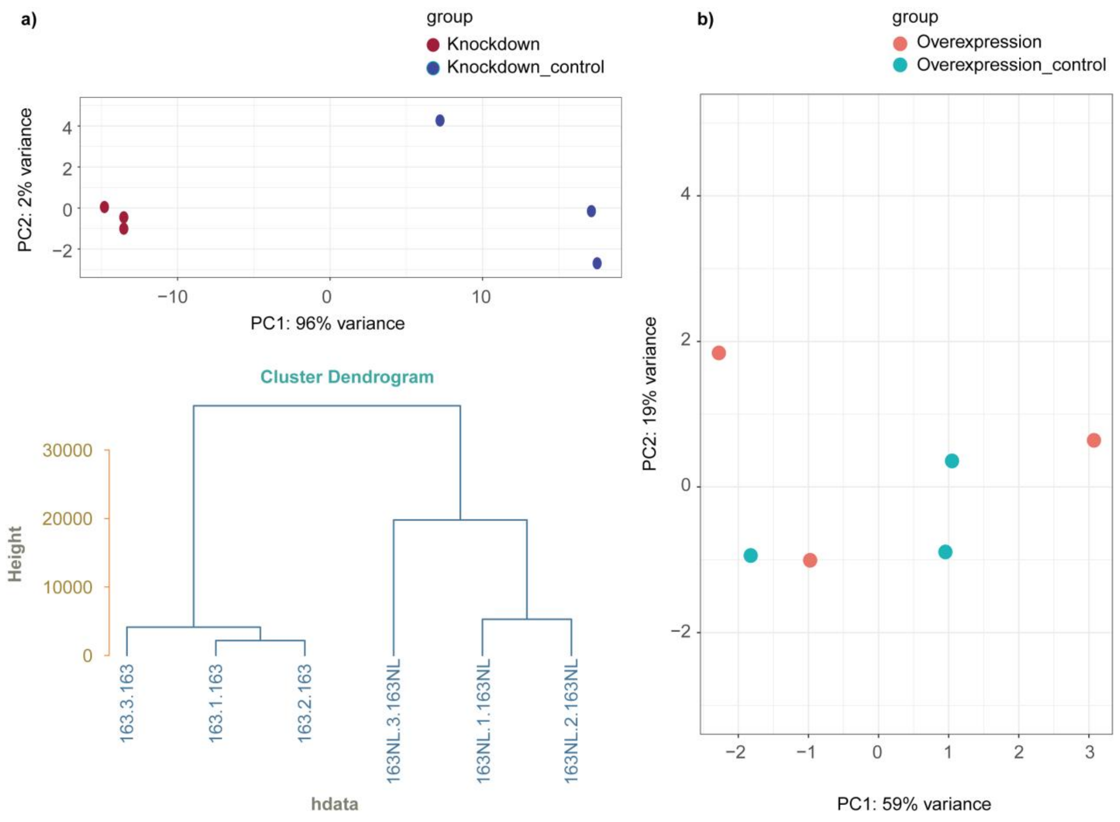

Supplementary Figure 8. Clustering of *HSALNG0104472* knockdown and overexpression samples. PCA and hierarchical clustering were performed to *HSALNG0104472* knockdown **a)** and overexpression **b)** samples (n = 3) compared to control group (n = 3) in AC16 cell lines. The colors of dots represent different group. It reveals that *HSALNG0104472* knockdown samples were clustered well, while overexpression of *HSALNG0104472* did not show significant effect on gene expression in AC16 cells. The knockdown and overexpression experiments were conducted in three biological replicates, respectively.

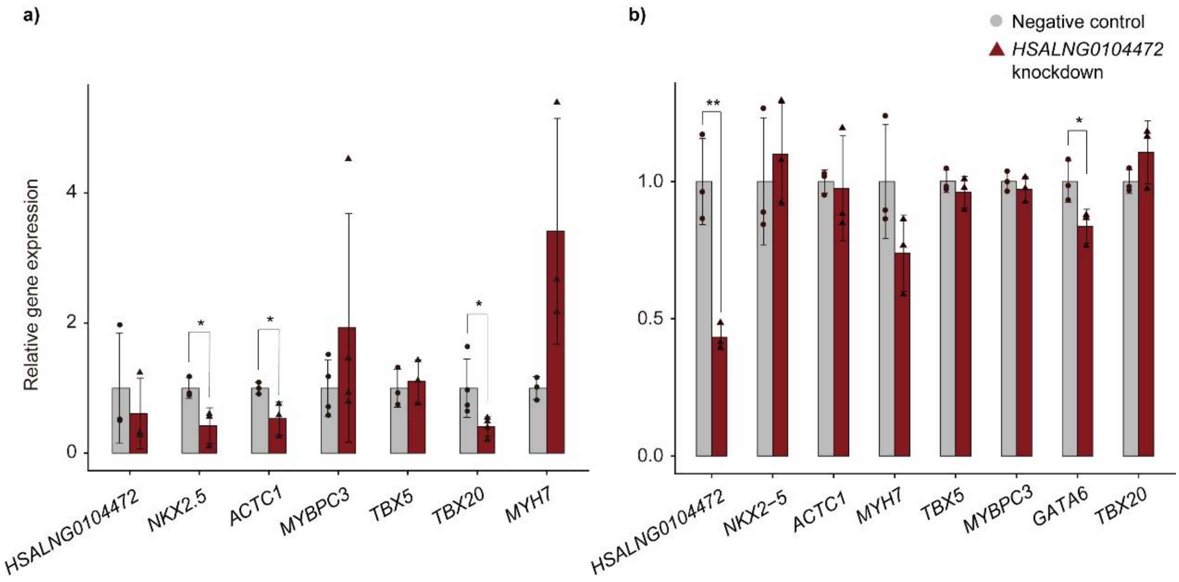

Supplementary Figure 9. qPCR analyses of *HSALNG0104472* knockdown iPSC and iPSC-cardiomyocytes. a) qPCR analysis was used to detect expression changes for CHD genes that were coexpressed with *HSALNG0104472* in *HSALNG0104472* knockdown human induced pluripotent stem cells (iPSCs). **b)** qPCR analysis was used to detect expression changes for CHD genes that were coexpressed with *HSALNG0104472* after *HSALNG0104472* knockdown in induced pluripotent stem cell derived cardiomyocytes (hiPSC-CMs). The grey and dark red bars represent the *HSALNG0104472* knockdown and negative control (NC) groups, respectively. The knockdown experiments were conducted in at least three biological replicates (Supplementary Data 13). The error bars are shown as means ± SD. Two-tailed t test was used for comparison between two groups. *$P < 0.05$, **$P < 0.01$.

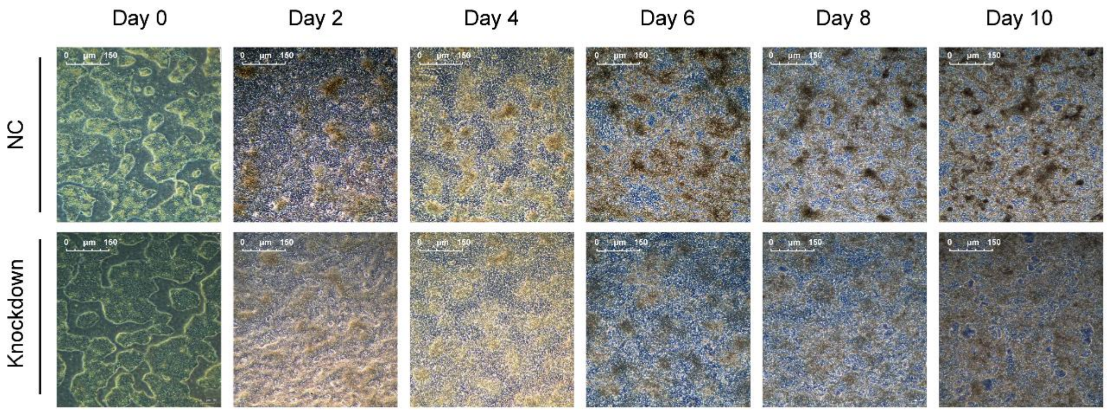

Supplementary Figure 10. Differentiation of *HSALNG0104472* knockdown human iPSCs to cardiomyocytes. For *HSALNG0104472* knockdown and control groups, 6 time points (day 0, 2, 4, 6, 8, 10 post induction) during the differentiation of human iPSCs to cardiomyocytes were captured. Scale bar, 150 μm.

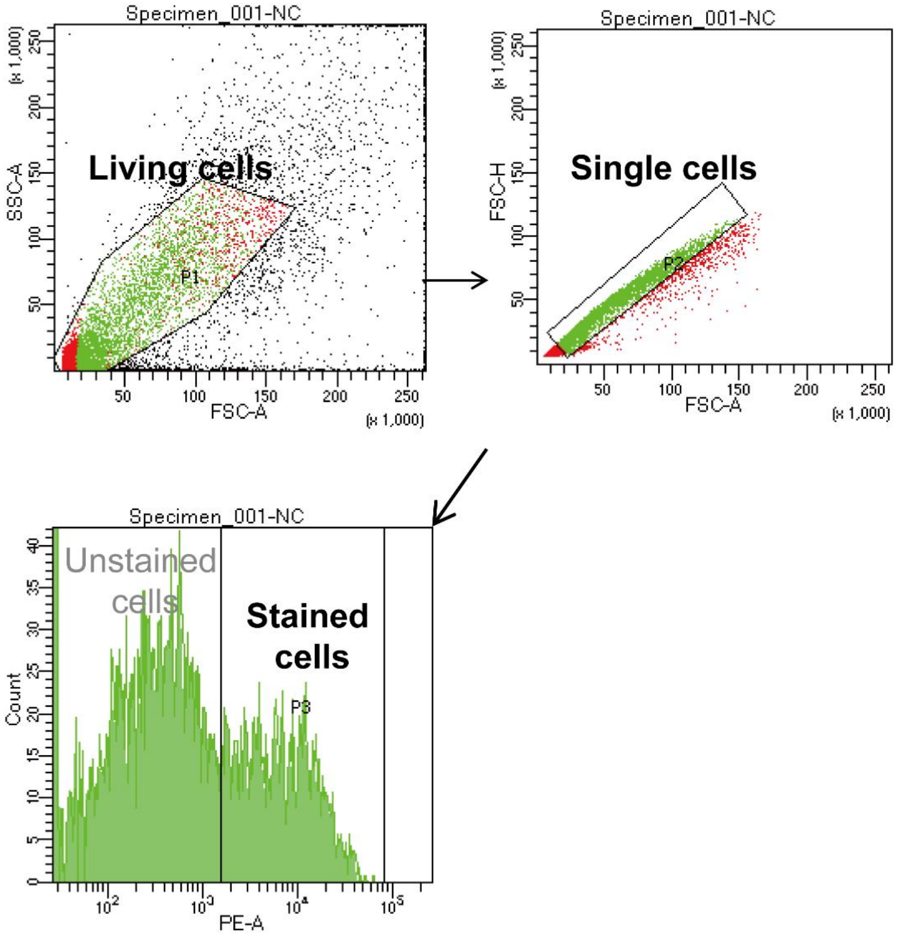

Tube: NC

Population	#Events	%Parent	%Total
All Events	10,000	####	100.0
P1	7,180	71.8	71.8
P2	3,911	54.5	39.1
P3	1,188	30.4	11.9

Supplementary Figure 11. Reduction of CNV-lncRNA *HSALNG0104472* may affect the efficiency of cardiomyocyte differentiation (Control group 1, including gating strategy). Flow cytometry for evaluating the relative yield of cardiomyocytes and efficiency of cell differentiation by measuring the number of cells (at day 8) expressing cardiac-specific proteins cardiac Troponin T (cTnT). Three biological replicates were conducted for *HSALNG0104472* knockdown and control group (A supplement to Fig. 8b, Supplementary Data 13). Gating strategy was shown. NC: control group 1.

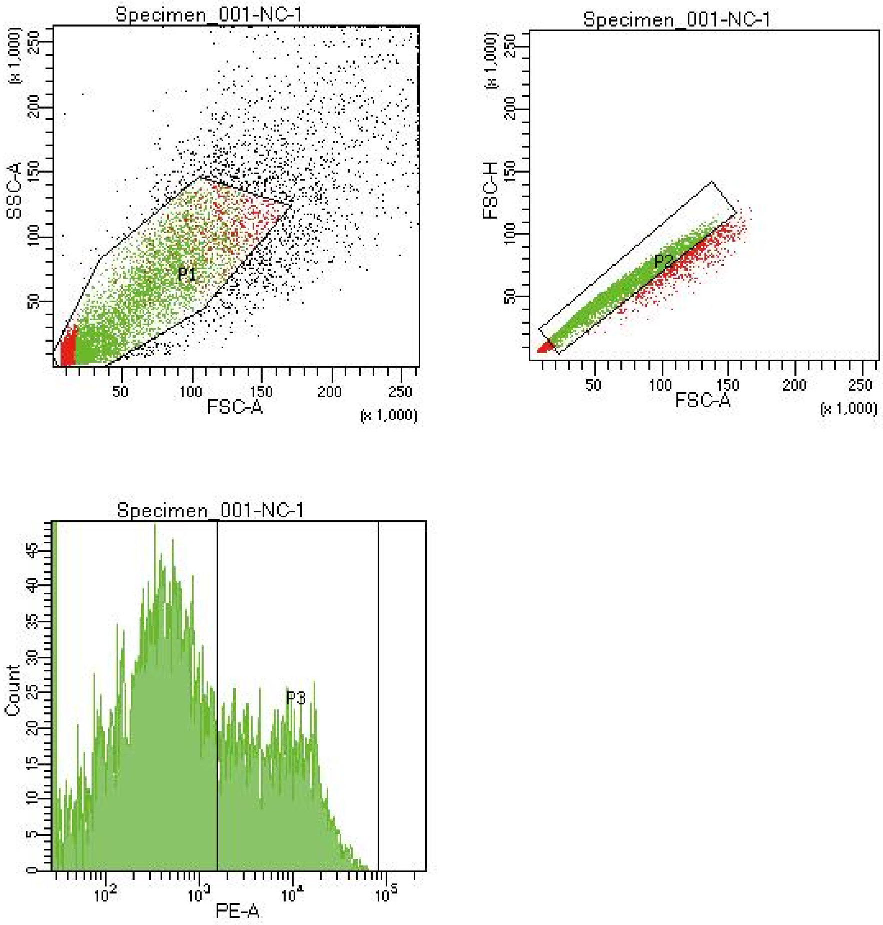

Tube: NC-1

Population	#Events	%Parent	%Total
All Events	10,000	####	100.0
P1	7,209	72.1	72.1
P2	4,379	60.7	43.8
P3	1,402	32.0	14.0

Supplementary Figure 12. Reduction of CNV-lncRNA *HSALNG0104472* may affect the efficiency of cardiomyocyte differentiation (Control group 2). Flow cytometry for evaluating the relative yield of cardiomyocytes and efficiency of cell differentiation by measuring the number of cells (at day 8) expressing cardiac-specific proteins cardiac Troponin T (cTnT). Three biological replicates were conducted for *HSALNG0104472* knockdown and control group (A supplement to Fig. 8b, Supplementary Data 13). Gating strategy was shown in Supplementary Figure 11. NC-1: control group 2.

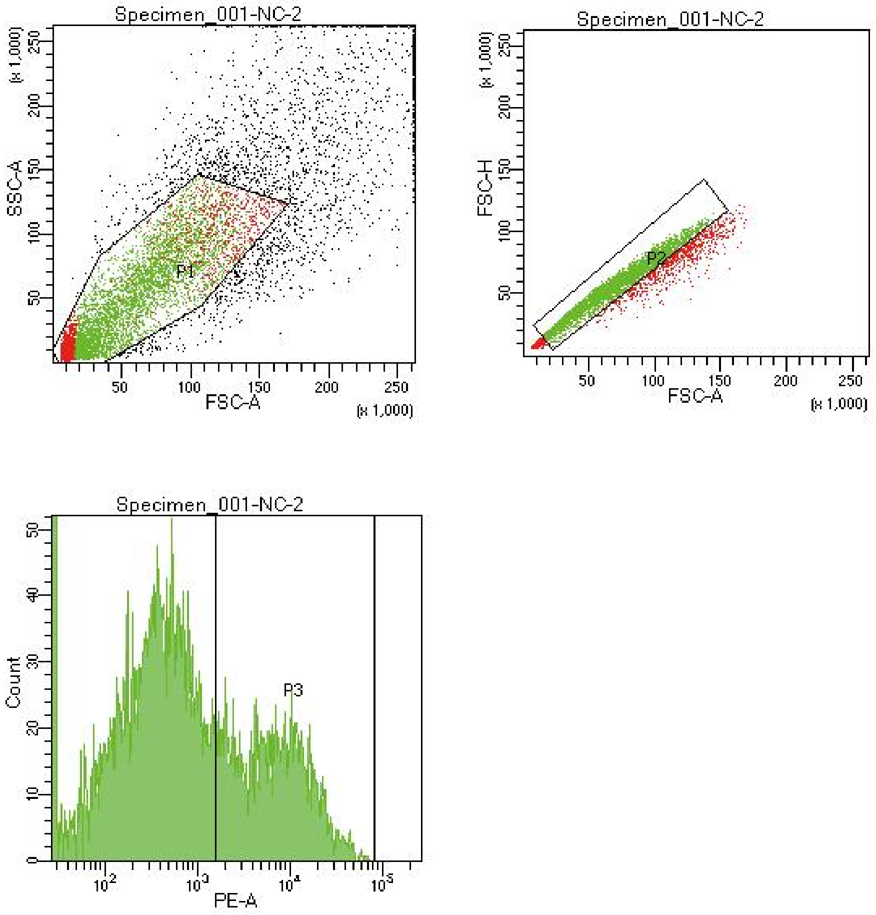

Tube: NC-2

Population	#Events	%Parent	%Total
All Events	10,000	####	100.0
P1	6,846	68.5	68.5
P2	4,113	60.1	41.1
P3	1,304	31.7	13.0

Supplementary Figure 13. Reduction of CNV-lncRNA *HSALNG0104472* may affect the efficiency of cardiomyocyte differentiation (Control group 3). Flow cytometry for evaluating the relative yield of cardiomyocytes and efficiency of cell differentiation by measuring the number of cells (at day 8) expressing cardiac-specific proteins cardiac Troponin T (cTnT). Three biological replicates were conducted for *HSALNG0104472* knockdown and control group (A supplement to Fig. 8b, Supplementary Data 13). Gating strategy was shown in Supplementary Figure 11. NC-2: control group 3.

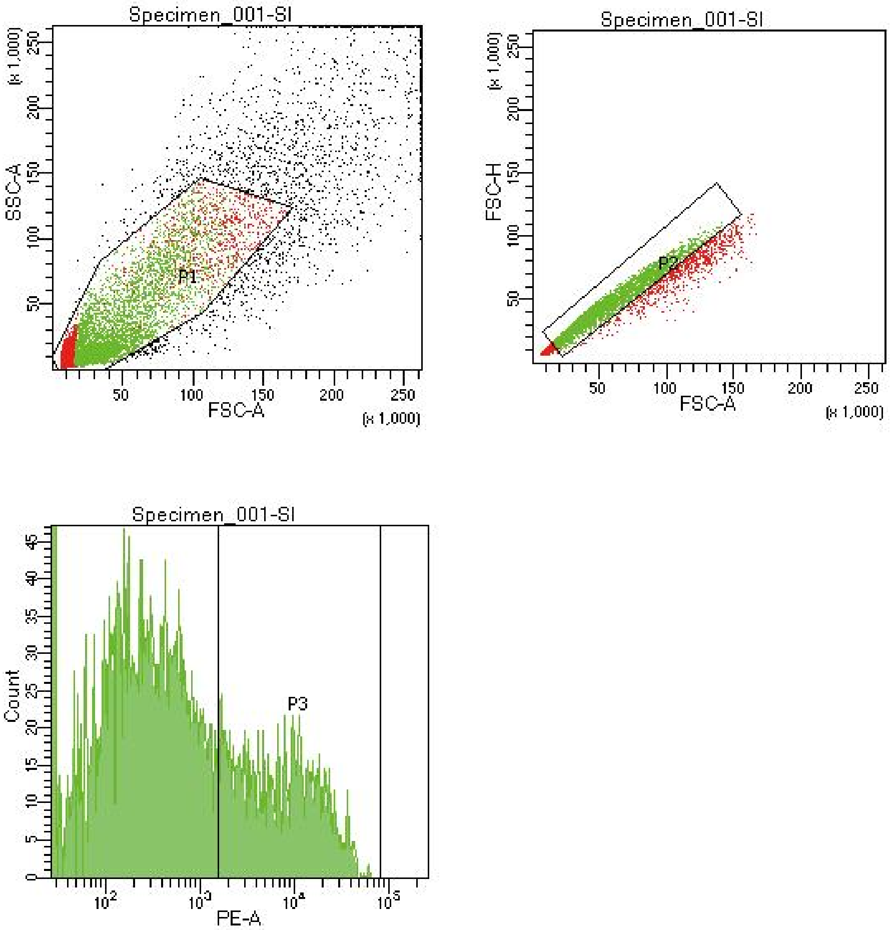

Tube: SI

Population	#Events	%Parent	%Total
All Events	10,000	####	100.0
P1	7,717	77.2	77.2
P2	4,282	55.5	42.8
P3	1,172	27.4	11.7

Supplementary Figure 14. Reduction of CNV-lncRNA *HSALNG0104472* may affect the efficiency of cardiomyocyte differentiation (*HSALNG0104472* knockdown group 1). Flow cytometry for evaluating the relative yield of cardiomyocytes and efficiency of cell differentiation by measuring the number of cells (at day 8) expressing cardiac-specific proteins cardiac Troponin T (cTnT). Three biological replicates were conducted for *HSALNG0104472* knockdown and control group (A supplement to Fig. 8b, Supplementary Data 13). Gating strategy was shown in Supplementary Figure 11. SI: *HSALNG0104472* knockdown group 1.

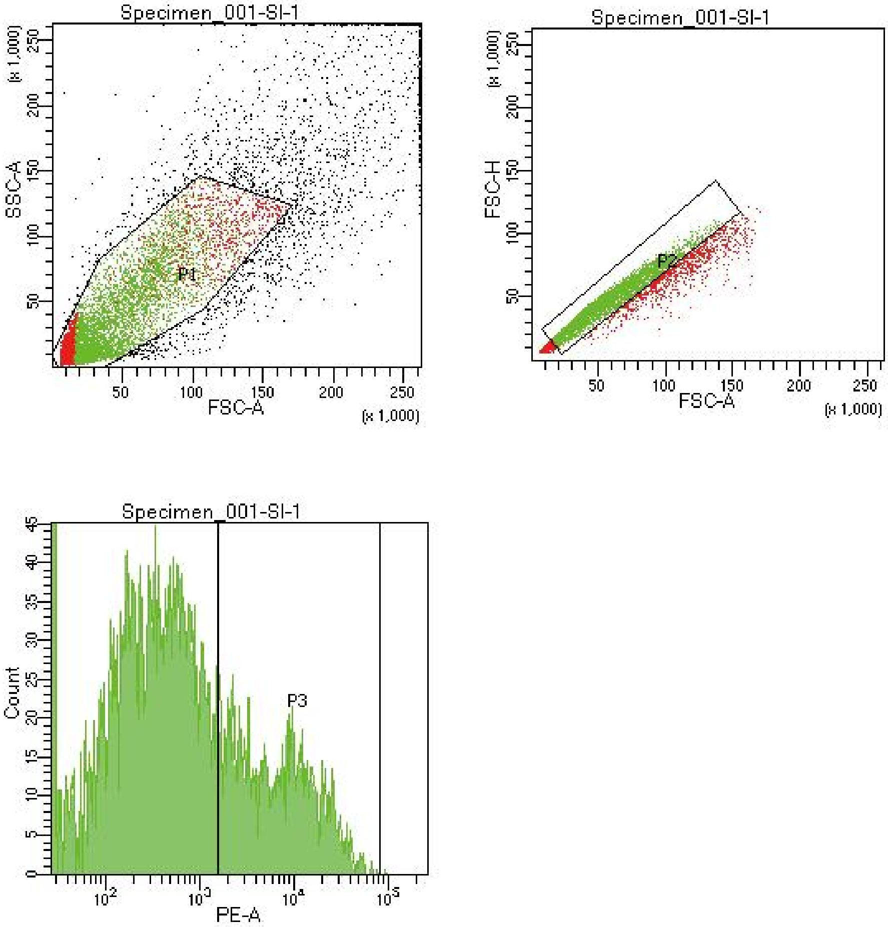

Tube: SI-1

Population	#Events	%Parent	%Total
All Events	10,000	####	100.0
P1	7,623	76.2	76.2
P2	4,161	54.6	41.6
P3	1,139	27.4	11.4

Supplementary Figure 15. Reduction of CNV-lncRNA *HSALNG0104472* may affect the efficiency of cardiomyocyte differentiation (*HSALNG0104472* knockdown group 2). Flow cytometry for evaluating the relative yield of cardiomyocytes and efficiency of cell differentiation by measuring the number of cells (at day 8) expressing cardiac-specific proteins cardiac Troponin T (cTnT). Three biological replicates were conducted for *HSALNG0104472* knockdown and control group (A supplement to Fig. 8b, Supplementary Data 13). Gating strategy was shown in Supplementary Figure 11. SI-1: *HSALNG0104472* knockdown group 2.

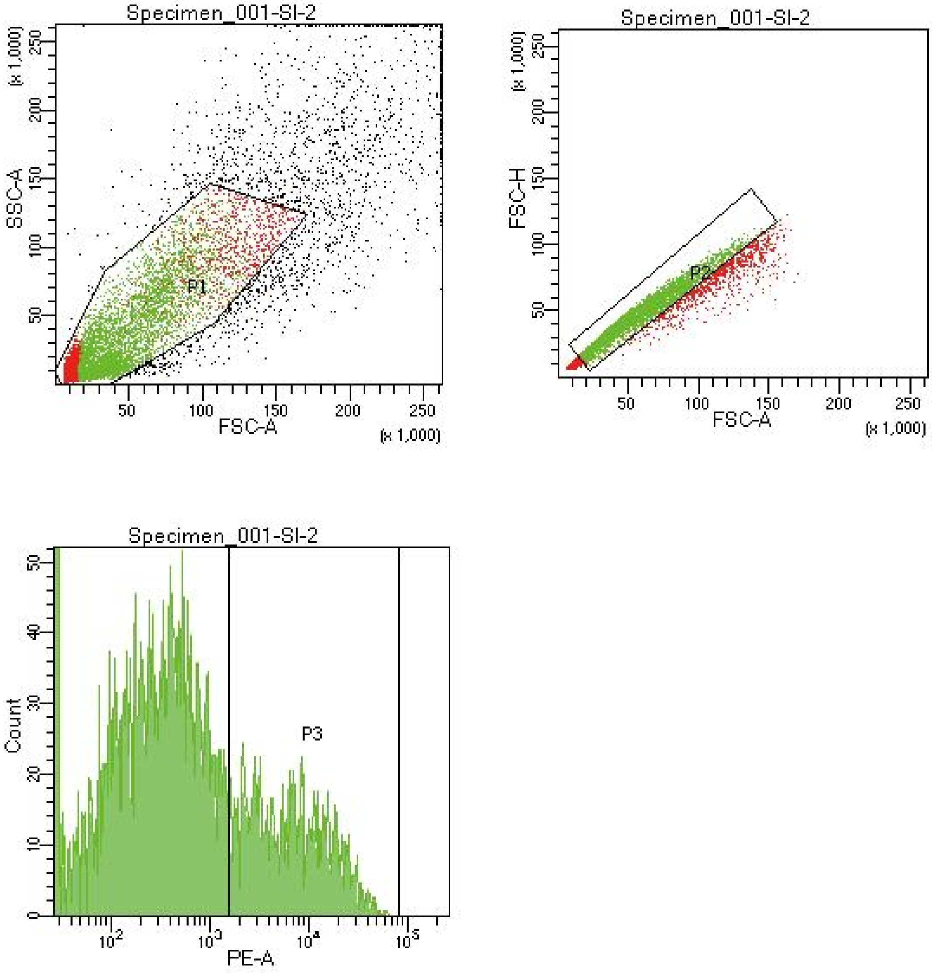

Tube: SI-2

Population	#Events	%Parent	%Total
All Events	10,000	####	100.0
P1	7,844	78.4	78.4
P2	4,274	54.5	42.7
P3	1,077	25.2	10.8

Supplementary Figure 16. Reduction of CNV-lncRNA *HSALNG0104472* may affect the efficiency of cardiomyocyte differentiation (*HSALNG0104472* knockdown group 3). Flow cytometry for evaluating the relative yield of cardiomyocytes and efficiency of cell differentiation by measuring the number of cells (at day 8) expressing cardiac-specific proteins cardiac Troponin T (cTnT). Three biological replicates were conducted for *HSALNG0104472* knockdown and control group (A supplement to Fig. 8b, Supplementary Data 13). Gating strategy was shown in Supplementary Figure 11. SI-2: *HSALNG0104472* knockdown group 3.

Supplementary Table

Supplementary Table 1. Abbreviation and corresponding full names of CHD type in Table 1.

Abbreviation	Full Name
TGA	transposition of the great arteries
ASD	atrial septal defect
MV	mechanical ventilation
VSD	ventricular septal defect
PDA	patent ductus arteriosus
TOF	tetralogy of Fallot
PA	pulmonary atresia
APV	absent pulmonary valve syndrome
CTD	conotruncal defects
LS-CHD	left-sided congenital heart disease
RLBV	aretroesophageal left brachiocephalic vein
DORV	double outlet right ventricle
CoA	coarctation of aorta
LSVC	left superior vena cava
MA	mitral valve atresia
CSD	coronary sinus dilation
CIAV	congenital insufficiency of aortic valve
PFO	patent foramen ovale
BAV	bicuspid aortic valve
AI	aortic insufficiency
ALV	abnormal lung vessels
TAPVC	total abnormal pulmonary venous connection
LVOT	left ventricular outflow tract obstruction
TAPVR	total anomalous pulmonary venous return
D-TGA	dextro transposition of the great arteries
AS	aortic stenosis
PS	pulmonary stenosis
ECD	endocardial cushion defect
MAPCA	major aorto pulmonary collateral artery
IAA	interrupted aortic arch
PTA	persistent truncus arteriosus
AVS	aortic valve stenosis
HRH	hypoplastic right heart syndrome
RAA	right aortic arch
BSVC	bilateral superior vera cava
TA	tricuspid atresia
AC	aortic coarctation
SA	single atrium
PAPVC	partial anomaly of pulmonary venous connection
SAS	subvalvular aortic stenosis
TVD	tricuspid valve disease
RVH	right ventricular hypoplasia
EA	Ebstein's anomaly
SV	single ventricular
DOA	dilatation of aorta

Supplementary Table 2. 21 CNVs associated with syndromic CHD.

CNV location	CNV type	Related syndrome	Related CHD-risk genes	Reference[2]
1p36	deletion	1p36 Deletion Syndrome		Zhang, Xun et al. 2021
1q21.1	deletion /duplication	1q21.1 deletion/duplication		Bernier et al. 2016
1q41	deletion	1q41-q42 deletion		Rosenfeld et al. 2011
1q42	deletion	1q41-q42 deletion		Rosenfeld et al. 2011
1q43	deletion	1q43-q44 deletion		van Bon et al. 2008
1q44	deletion	1q43-q44 deletion		van Bon et al. 2008
2q31.1	deletion	2q31.1 deletion		Mitter et al. 2010
2q37.3	deletion	2q37 deletion		Casas et al. 2004
7q11.23	deletion	Williams–Beuren syndrome		Eronen M et al. 2002
8p23.1	deletion	8p23.1 deletion	*GATA4*	Wat MJ et al. 2009
9q34.3	deletion	Kleefstra syndrome		Kleefstra et al. 2006
11q24.3	deletion	Jacobsen syndrome		Favier R et al. 2015
15q24	deletion	15q24 deletion		Mefford et al. 2012
16p11.2	deletion	16p11.2p-12.2 deletion		Hempel M et al. 2009
16p12.1	deletion	16p11.2p-12.2 deletion		Hempel M et al. 2009
16p12.2	deletion	16p11.2p-12.2 deletion		Hempel M et al. 2009
17q21.31	deletion	Koolen–de Vries syndrome		Koolen et al. 2008
20p12	deletion	Alagille syndrome	*JAG1*	McElhinney DB et al. 2002
20p13	deletion	Alagille syndrome	*JAG1*	McElhinney DB et al. 2002
22q11.21	deletion /duplication	22q11.2 deletion syndrome (DiGeorge, velocardiofacial syndrome)	*TBX1*	Ou, Zhishuo et al. 2008
22q13.3	deletion	Phelan–McDermid syndrome	*SHANK3*	Phelan K et al. 2012

Supplementary Table 3. The silencer sequences for transient transfection in *HSALNG0104472* knockdown experiments.

The silencer sequences
GAACTGCCTGAATTGCTGCA
TAAGGAACGCTGGCAAAGGA
AAGCTGTGCAAGCAGATGGA
CTAGATGAGTCATTAAGGT
GTTGGATGAAGAAGAAGCA
GAACCGTATAAGCCTTTAA

Supplementary Table 4. Primers for quantitative reverse transcription qPCR analyses.

	**Primer1**	**Primer2**
GAPDH	GGAGCGAGATCCCTCCAAAAT	GGCTGTTGTCATACTTCTCATGG
ACTC1	TCCCATCGAGCATGGTATCAT	GGTACGGCCAGAAGCATACA
MYBPC3	CATGAGGCGCGATGAGAAGA	AAAGATGTACTTGCTGCCGCT
TBX5	TTGTGTTCTTTGGCGCTTGC	CGCAAGGTTCTGCTCTCGTT
MYH7	CTGCTCTGTGTCTTTCCCTGCT	CCTGTTTGTCATCAGGCACGA
TBX20	GCATTCCTATGCACGCTCAC	TGTAAAGGCTGACCCTCGAT
GATA6	CTCAGTTCCTACGCTTCGCAT	GTCGAGGTCAGTGAACAGCA
NKX2-5	CCAAGGACCCTAGAGCCGAA	ATAGGCGGGGTAGGCGTTAT

Supplementary References

1 Li, Z. *et al.* LncBook 2.0: integrating human long non-coding RNAs with multi-omics annotations. *Nucleic Acids Res*, doi:10.1093/nar/gkac999 (2022).

2 Pierpont, M. E. *et al.* Genetic Basis for Congenital Heart Disease: Revisited: A Scientific Statement From the American Heart Association. *Circulation* **138**, e653-e711, doi:10.1161/CIR.0000000000000606 (2018).
