# Supplementary material for: Copy number variation-associated lncRNAs may contribute to the etiologies of congenital heart disease
Source: Commun Biol. 2023 Feb 17;6:189. doi: 10.1038/s42003-023-04565-z (PMC9938258; doi:10.1038/s42003-023-04565-z)
Supplement: Supplementary file 3 — Description of Additional Supplementary Files [file 42003_2023_4565_MOESM3_ESM.pdf]

Description of Additional Supplementary Files

File name: Supplementary Data 1 (.xlsx).

Description: CHD associated CNV-lncRNAs and

WGCNA.

File name: Supplementary Data 2 (.xlsx).

Description: Pathway analysis of modules correlated to

sample traits.

File name: Supplementary Data 3 (.xlsx).

Description: Sequence conservation of CNV-lncRNAs

and HSALNG0104472 in 40 vertebrates.

File name: Supplementary Data 4 (.xlsx).

Description: Intersection of CNV-lncRNAs and nonsyndromic CHD associated CNVs.

File name: Supplementary Data 5 (.xlsx).

Description: Enrichment analyses of genes in

coexpression modules against 4 CHD-related gene sets.

File name: Supplementary Data 6 (.xlsx).

Description: CeRNA regulatory networks mediated by

CNV-lncRNAs.

File name: Supplementary Data 7 (.xlsx).

Description: DEGs between heart and brain samples in

human organ developmental transcriptomic dataset.

File name: Supplementary Data 8 (.xlsx).

Description: Summary of WGCNA over nonsyndromic and syndromic CHD associated CNVlncRNAs.

File name: Supplementary Data 9 (.xlsx).

Description: Enrichment analyses of genes in

coexpression modules against gene set related to autism

spectrum disorder.

File name: Supplementary Data 10 (.xlsx).

Description: Relative weight analysis of regulatory role

of CNV-lncRNAs to coexpressed CHD genes.

File name: Supplementary Data 11 (.xlsx).

Description: Expression pattern of 30 CNV-lncRNAs and 12 coexpressed CHD genes in heart-related black module (non-syndromic) in cardiomyocyte differentiation dataset.

File name: Supplementary Data 12 (.xlsx).

Description: RNA-seq analyses of HSALNG0104472 knockdown and overexpression adult human cardiomyocyte (AC16).

File name: Supplementary Data 13 (.xlsx).

Description: qPCR analyses of HSALNG0104472 in human induced pluripotent stem cell-derived cardiomyocytes (hiPSC-CMs) and cardiomyocytes.

File name: Supplementary Movie 1 (.mov).

Description: The beating behavior of differentiated HSALNG0104472 knockdown cardiomyocytes.

File name: Supplementary Movie 2 (.mov).

Description: The beating behavior of differentiated control cardiomyocytes.
