# Supplementary material for: Copy number variation-associated lncRNAs may contribute to the etiologies of congenital heart disease
Source: Commun Biol. 2023 Feb 17;6:189. doi: 10.1038/s42003-023-04565-z (PMC9938258; doi:10.1038/s42003-023-04565-z)
Supplement: Supplementary file 19 — Reporting Summary [file 42003_2023_4565_MOESM19_ESM.pdf]

nature portfolio

Corresponding author(s): Bo Wang

Last updated by author(s): Jan 31, 2023

Reporting Summary

Nature Portfolio wishes to improve the reproducibility of the work that we publish. This form provides structure for consistency and transparency in reporting. For further information on Nature Portfolio policies, see our Editorial Policies and the Editorial Policy Checklist.

Statistics

For all statistical analyses, confirm that the following items are present in the figure legend, table legend, main text, or Methods section.

n/a	Confirmed	
☐	☒	The exact sample size (*n*) for each experimental group/condition, given as a discrete number and unit of measurement
☐	☒	A statement on whether measurements were taken from distinct samples or whether the same sample was measured repeatedly
☐	☒	The statistical test(s) used AND whether they are one- or two-sided<br>*Only common tests should be described solely by name; describe more complex techniques in the Methods section.*
☒	☐	A description of all covariates tested
☒	☐	A description of any assumptions or corrections, such as tests of normality and adjustment for multiple comparisons
☐	☒	A full description of the statistical parameters including central tendency (e.g. means) or other basic estimates (e.g. regression coefficient) AND variation (e.g. standard deviation) or associated estimates of uncertainty (e.g. confidence intervals)
☐	☒	For null hypothesis testing, the test statistic (e.g. *F*, *t*, *r*) with confidence intervals, effect sizes, degrees of freedom and *P* value noted<br>*Give P values as exact values whenever suitable.*
☒	☐	For Bayesian analysis, information on the choice of priors and Markov chain Monte Carlo settings
☐	☒	For hierarchical and complex designs, identification of the appropriate level for tests and full reporting of outcomes
☐	☒	Estimates of effect sizes (e.g. Cohen's *d*, Pearson's *r*), indicating how they were calculated

Our web collection on statistics for biologists contains articles on many of the points above.

Software and code

Policy information about availability of computer code

Data collection	No software was used.
Data analysis	The R package WGCNA v1.70 was used for coexpression network construction and module identification. The R package clusterProfiler v3.10 was used for Gene ontology (GO) enrichment analysis and Kyoto Encyclopedia of Genes and Genomes (KEGG) pathway enrichment analysis. The R package DESeq2 v1.32 was used for differential expression analysis. BEDTools v2.29.2 was used for CNV-lncRNA retrieval. LncmiRSRN v3.0 was used for lncRNA-miRNA-mRNA regulatory network analysis. For RNAseq analysis, the raw data was processed with following tools: quality analysis and base quality filtering with FastQC v0.11.9 and Trim Galore v0.6.6, rRNA removing with SortMeRNA v4.3.4, alignment with STAR v2.7.10, reads counting with featureCounts implemented in the Subread package v2.0.3. All citations of these tools are included in main text and supplementary materials.

For manuscripts utilizing custom algorithms or software that are central to the research but not yet described in published literature, software must be made available to editors and reviewers. We strongly encourage code deposition in a community repository (e.g. GitHub). See the Nature Portfolio guidelines for submitting code & software for further information.

Data

Policy information about availability of data

All manuscripts must include a data availability statement. This statement should provide the following information, where applicable:

- Accession codes, unique identifiers, or web links for publicly available datasets
- A description of any restrictions on data availability
- For clinical datasets or third party data, please ensure that the statement adheres to our policy

The RNAseq raw data for overexpression and knockdown experiments on HSALNG0104472 in AC16 cardiomyocytes that support the findings of this study are openly available in GEO: GSE201076. All data are available in the main text or the supplementary materials.

Human research participants

Policy information about studies involving human research participants and Sex and Gender in Research.

Reporting on sex and gender	No human participant was involved in this study.
Population characteristics	n/a
Recruitment	n/a
Ethics oversight	n/a

Note that full information on the approval of the study protocol must also be provided in the manuscript.

Field-specific reporting

Please select the one below that is the best fit for your research. If you are not sure, read the appropriate sections before making your selection.

☒ Life sciences ☐ Behavioural & social sciences ☐ Ecological, evolutionary & environmental sciences

For a reference copy of the document with all sections, see nature.com/documents/nr-reporting-summary-flat.pdf

Life sciences study design

All studies must disclose on these points even when the disclosure is negative.

Sample size	Sample size was deemed to be appropriate based on the fact that Meng et al. were able to detect coexpression relationships between CNV-lncRNAs and SCZ-related genes in similar studies, previously published, involving smaller datasets. The coexpression relationships calculated in present study with larger sample sizes were deemed to be robust.
Data exclusions	Pre-established QC criteria were applied to the data (e.g. gene expression value in heart samples).
Replication	Findings on correlations between CNV-lncRNAs and key CHD genes were replicated in the human organ development dataset, cardiomyocyte differentiation dataset and in vitro experiments in cardiomyocyte cell lines (AC16) , induced pluripotent stem cells (iPSCs) and differentiated cardiomyocytes.
Randomization	n/a
Blinding	n/a

Reporting for specific materials, systems and methods

We require information from authors about some types of materials, experimental systems and methods used in many studies. Here, indicate whether each material, system or method listed is relevant to your study. If you are not sure if a list item applies to your research, read the appropriate section before selecting a response.

Materials & experimental systems

n/a	Involved in the study
☐	☒ Antibodies
☐	☒ Eukaryotic cell lines
☒	☐ Palaeontology and archaeology
☒	☐ Animals and other organisms
☒	☐ Clinical data
☒	☐ Dual use research of concern

Methods

n/a	Involved in the study
☒	☐ ChIP-seq
☐	☒ Flow cytometry
☒	☐ MRI-based neuroimaging

Antibodies

Antibodies used

cTnI (RRID: AB_2532494, ThermoFisher, Waltham, MA, USA), α-Actinin (RRID: AB_2692251, ThermoFisher, Waltham, MA, USA), Cardiac Troponin T Polyclonal Antibody PE Conjugated (Catalog No: #C90559PE)

Validation

All antibodies were validated by the manufacturer.

Eukaryotic cell lines

Policy information about cell lines and Sex and Gender in Research

Cell line source(s)

HEK293 (#sKO334, Cyagen, Suzhou, China), AC16 (#GDCELL-0591, Cellbio, Shanghai, China) , iPSC (established previously in our lab: Li X. et al., J Med Genet. 2021. doi:10.1136/jmedgenet-2019-106608)

Authentication

Morphology or genotyping.

Mycoplasma contamination

Cells tested negative for mycoplasma contamination by the commcercial vendor. Cells were not tested after receipt.

Commonly misidentified lines
(See ICLAC register)

No commonly misidentified lines were used.

Flow Cytometry

Plots

Confirm that:

☒ The axis labels state the marker and fluorochrome used (e.g. CD4-FITC).

☒ The axis scales are clearly visible. Include numbers along axes only for bottom left plot of group (a 'group' is an analysis of identical markers).

☒ All plots are contour plots with outliers or pseudocolor plots.

☒ A numerical value for number of cells or percentage (with statistics) is provided.

Methodology

Sample preparation

Digest cardiomyocytes with mild digestive enzymes for 5 min. Add an equal volume of cell culture medium to stop the reaction. Centrifuge at 25? 800×g for 5 minutes and resuspend cells with PBS to wash the cells 2 times at the same procedure. Aliquot 1 x 10^6 cells from pretreated sample in 100 ul by volume in a 5 ml assay tube per test. Add Cardiac Troponin T Polyclonal Antibody PE Conjugated at the appropriate dilution (1:100) to the assay tubes. Incubate at 37? for 1 hour. Wash by centrifugation in 2-3 ml wash buffer (PBS with 0.1% FBS). Resuspend cells in 0.3-0.5 ml PBS and analyze on flow cytometer.

Instrument

BD FACSCanto™ flow cytometer was used for cardiomyocyte differentiation assays.

Software

The percentage of cells was assessed by BD FACSDiva™ v8.0.1 Software.

Cell population abundance

Abundance of positive and negative fractions were determined by cell counting post sort.

Gating strategy

Gating strategy was shown in Supplementary Information (Supplementary Figure 10).

☒ Tick this box to confirm that a figure exemplifying the gating strategy is provided in the Supplementary Information.
